# Detection and genetic characterization of “*Candidatus Mycoplasma* haemomacaque” infection among long-tailed macaques (*Macaca fascicularis*) in Thailand using broad-range nested polymerase chain reaction assay

**DOI:** 10.14202/vetworld.2021.943-948

**Published:** 2021-04-19

**Authors:** Wanat Sricharern, Supakarn Kaewchot, Sarawan Kaewmongkol, Natnaree Inthong, Thitichai Jarudecha, Rucksak Rucksaken, Bandid Mangkit, Sakulchit Wichianchot, Tawin Inpankaew

**Affiliations:** 1 Center for Agricultural Biotechnology, Kasetsart University, Kamphaeng Saen Campus, Nakhon Pathom, Thailand; 2 Center of Excellence on Agricultural Biotechnology, Science and Technology Postgraduate Education and Research Development Office, Commission on Higher Education, Ministry of Education, Science, Research Innovation (AG-BIO/PERDO-CHE), Bangkok, Thailand; 3 Department of Veterinary Technology, Faculty of Veterinary Technology, Kasetsart University, Bangkok, Thailand; 4 Department of National Park, Wildlife and Plant Conservation, Bangkok, Thailand; 5 Department of Parasitology, Faculty of Veterinary Medicine, Kasetsart University, Bangkok, Thailand

**Keywords:** broad-range nested polymerase chain reaction, hemoplasma, long-tailed macaque, mycoplasma, Thailand

## Abstract

**Background and Aim:**

Hemoplasmas are defined as small, epicellular parasitic bacteria that can infect the red blood cells of several mammalian species. Diseases caused by these bacteria range from asymptomatic infections to acute hemolytic anemia. However, data on hemoplasmas in non-human primates in Thailand remain to be limited. Therefore, this study aims to determine the occurrence and genetic diversity of hemoplasmas among long-tailed macaques in Thailand.

**Materials and Methods:**

Blood samples were collected from 339 long-tailed macaques in three provinces of Thailand. DNA was then extracted from the blood samples and tested for hemoplasma using broad-range nested polymerase chain reaction (PCR) based on the *16S rRN*A gene. PCR-positive samples were sequenced, and phylogenetic analysis for species identification was conducted.

**Results:**

In total, 38 (11.2%) out of the 339 samples were found to be positive for hemoplasmas, based on the broad-range nested PCR assay of the *16S rRNA* gene. The *16S rRNA* sequences of *Mycoplasma* spp. were highly similar (98-99% identity) to “*Candidatus Mycoplasma* haemomacaque.” Furthermore, phylogenetic analysis using maximum likelihood demonstrated that the sequences were located in the same cluster of “*Ca. M*. haemomacaque.”

**Conclusion:**

The detection of hemoplasmas among long-tailed macaques in Thailand is reported. Genetic characterization confirmed that these hemoplasmas are closely related to “*Ca. M*. haemomacaque.” These results indicate that long-tailed macaques in several locations in Thailand may be infected and serve as reservoirs for this parasite.

## Introduction

Hemotropic *Mycoplasma* spp., also referred to as hemoplasmas, are Gram-negative, obligate, parasitic exoerythrocytic bacteria, which are uncultivable and are known to have no cell wall [1,2]. Hemoplasmas cause various clinical signs, ranging from asymptomatic to life-threatening, depending on host susceptibility [1,2]. Acute disease is associated with massive parasitemia, causing severe and sometimes fatal hemolytic anemia and other clinical symptoms, including lethargy, anorexia, fever, and pale or icteric mucous membranes [1]. Hemoplasma DNA was detected in *Amblyomma* spp. and lice, which might be vital in the transmission of these bacteria [3]. Hemoplasma infections are often detected in a wide range of mammalian species, including humans [4,5], dogs [6,7], cats [8,9], pigs [10], rodents [3], sheep, goats [11], and water buffaloes [12]. Several species of hemoplasmas were also detected in non-human primates, including “*Candidatus* *Mycoplasma* kahanei” in squirrel monkeys (*Saimiri sciureus*) and howler monkeys [13,14]; “*Ca. M*. aoti” in owl monkeys (*Aotus trivirgatus*) [15]; “*Candidatus Mycoplasma* haemomacaque” in cynomolgus monkeys (*Macaca fascicularis*), rhesus monkeys (*Macaca mulatta*), and Japanese monkeys (*Macaca fuscata*) [16-18]; and *Mycoplasma* spp. in capuchin monkeys, black tamarin, and black howler monkeys [13,14,19]. Some infected monkeys develop only mild anemia, while others die from severe anemia [20].

Human hemoplasmas are rarely reported and are often suspected of coming from infected domestic animals [2], such as the *Mycoplasma haemofelis*-like infection reported in an HIV-positive patient in Brazil [4] and swine hemoplasmas reported in a farmworker in China [21]. The human hemoplasma species, that is, “*Ca. M*. haemohominis,” is distinct from veterinary hemoplasma species [5]. Moreover, potentially new species of hemoplasmas in wildlife, as or as a result of host shifts, have been reported [2]. For this reason, the investigation and monitoring of hemoplasma infections, particularly in animals that are closely associated with humans, are crucial.

Long-tailed macaques (*M. fascicularis*) are also called cynomolgus or crab-eating macaques. These macaques are widely distributed in the southern part of the Southeast Asian mainland [22]. In Thailand, there are many locations where long-tailed macaques live close to human communities and share the environment with humans. Thus, macaques might serve as reservoir hosts for abundant human pathogens, including hemotropic mycoplasmas. However, few studies regarding the prevalence and molecular characterization of hemoplasma among long-tailed macaques in Thailand have been conducted.

Thus, this study aimed to determine the molecular prevalence of hemoplasmas in free-ranging long-tailed macaques based on broad-range nested polymerase chain reaction (PCR), to genetically characterize the hemoplasma isolated from macaques, and to evaluate any associations between hemoplasma infection and the gender and locations of hosts.

## Materials and Methods

### Ethical approval

This research was approved by the Animal Ethics Committee of Kasetsart University, Bangkok, Thailand (ACKU59-VTN-004).

### Study period and location

The study was conducted during 2016 - 2018. The blood samples were collected from long-tailed macaques in Samut Songkhram, Chonburi, and Phuket provinces, Thailand, and the laboratory investigations were conducted at the Faculty of Veterinary Technology, Kasetsart University.

### Sample collection and study areas

**Figure-1:**
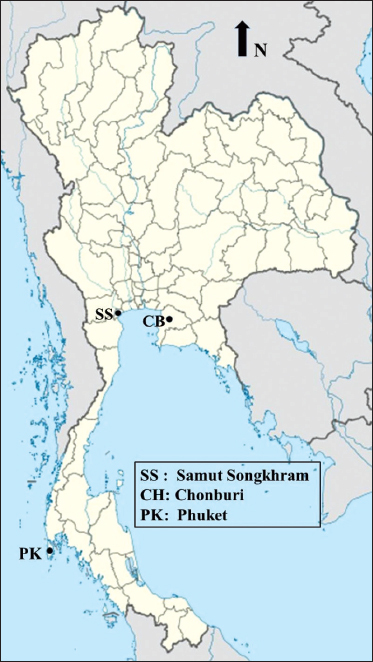
Locations of sampling sites. (https://commons.wikimedia.org/wiki/File:Thailand_location_map.svg).

Sample sizes were estimated using EpiTools (https://epitools.ausvet.com.au/oneproportion), with an estimated proportion of 0.3, based on the previous studies [19]. The precision of estimate and confidence level was set as 0.05 and 0.95, respectively. The calculated sample size was 323 samples. In this current study, 339 blood samples were collected for better representation and to account for rejected samples. In 2016-2018, blood samples were collected from free-ranging, long-tailed macaques inhabiting three locations adjacent to human communities in Thailand, including Samut Songkhram, Chonburi, and Phuket provinces. Macaques have been causing a lot of trouble near human residences in these areas (Figure-1). For each macaque, 2 mL of venous blood was collected in an EDTA tube. The samples were then transported on ice to the laboratory and stored at −40°C until further processing.

### Molecular analysis

DNA was extracted from 200 mL of each EDTA blood sample using an EZNA^®^ Tissue DNA Extraction Kit (OMEGA Bio-tek Inc.; USA), according to the manufacturer’s instructions. Extracted DNA samples were stored at −40°C until molecular analyses.

A previously described broad-range nested PCR protocol based on the *16S rRNA* gene [23] was used to amplify *Mycoplasma* spp. DNA using two sets of oligonucleotides. The primer sequences for the primary PCR were V1-F (5’-AGAGTTTGATCCTGGCTCAG-3’) and V9-R (5’-GNTACCTTGTTACGACTT-3’). The PCR mixture for primary PCR contained 1x PCR buffer, 2 mM MgCl_2_, 0.2 mM dNTPs, 1 mM of each primer, 0.04 U/mL Taq DNA polymerase, and 1 mL of DNA template; the volume was brought up to 25 mL with water. Cycling conditions were as follows: 5 min denaturation at 95°C, followed by 40 cycles of 95°C for 1 min, 50°C for 1 min, and 72°C for 1 min, and a final extension of 72°C for 10 min. The expected length of the primary PCR product was approximately 1400 bp. The primer sequences for the secondary PCR were V3-F (5’-ACTCCTACGGGAGGCAGCAG-3’) and V6-R (5’-CGACAGCCATGCANCACCT-3’). The PCR mixture for nested PCR contained 1x PCR buffer, 2 mM MgCl_2_, 0.2 mM dNTPs, 1 mM of each primer, 0.04 U/mL Taq DNA polymerase, and 1 mL of DNA template; the volume was brought up to 25 mL with water. The PCR cycling conditions were as follows: 95°C for 5 min, followed by 45 cycles of 95°C for 1 min, 55°C for 45 s, and 72°C for 45 s, and a final extension of 72°C for 10 min. The expected length of the nested PCR product was approximately 700 bp.

The PCR products were identified using electrophoresis on a 1.2% (w/v) agarose-TAE gel and visualized with ultraviolet transilluminator after staining the nucleic acids with GelStar^®^ (Cambrex BioScience; Rockland, USA). All PCR-positive samples with amplicons of the correct size were submitted for DNA purification and sequencing. The sequences were compared with published sequences in the GenBank nucleotide database using the BLAST program of the National Center for Biotechnology Information.

### Phylogenetic analysis

Nucleotide sequences from the *16S rRNA* gene of hemoplasma obtained from the samples together with reference sequences downloaded from GenBank were aligned using the BioEdit program version 7.5.2. The phylogenetic analysis of the nucleotide sequences was conducted using the maximum likelihood method based on the Kimura 2-parameter model in the Mega 7 software (http://www.megasoftware.net/). A bootstrap analysis was used to assess the robustness of the clusters using 1000 replicates.

### Statistical analysis

Statistical analysis was performed using multivariable logistic regression, odds ratio (OR), 95% confidence interval (95% CI), and p-values, to determine the associations between hemoplasma infection and host gender or host location. Results were considered significantly different when p<0.05.

## Results

### Prevalence of hemoplasma infection

PCR amplification of the *16S rRNA* gene fragments revealed that 11.2% (38/339; 95% CI: 8.0%-15.1%) of long-tailed macaques were positive for hemoplasma. The infection rate among male macaques was determined to be 11.2% (31/278; 95% CI: 7.7-15.4%), and the infection rate among female macaques was 11.5% (7/61; 95% CI: 4.7-22.2%). The infection rates from the Samut Songkhram, Chonburi, and Phuket samples were 8.0% (2/25; 95% CI: 1.0-26.0%), 9.6% (23/240; 95% CI: 6.2-14.0%), and 17.6% (13/74; 95% CI: 9.7-28.1%), respectively (Table-1). No significant associations were observed between hemoplasma infection rate and sex or location of macaques (Table-1).

**Table-1:** Collection details and numbers of hemoplasma-positive samples and odds ratio of hemoplasma infection between sex and location.

Variable	No. of animals	Positive n (%)	95% CI of proportion	OR	95% CI of OR	p*-*value
Sex						
Male	278	31 (11.2)	7.7-15.4	1		
Female	61	7 (11.5)	4.7-22.2	1.16	0.48-2.82	0.741
Location						
Chonburi	240	23 (9.6)	6.2-14.0	1		
Samut Songkhram	25	2 (8.0)	1.0-26.0	0.81	0.18-3.66	0.784
Phuket	74	13 (17.6)	9.7-28.1	2.04	0.97-4.30	0.060
Total	339	38 (11.2)	8.0-15.1		

OR=Odds ratio, CI=Confidence interval

DNA sequencing demonstrated that all hemoplasma sequences detected in the long-tailed macaques shared percentages of identity ranging from 98.94% to 99.84% with “*Ca. M*. haemomacaque.” “*Ca. M*. haemomacaque” was detected in cynomolgus macaques in the USA (KC512401) and Japanese macaques in Japan (AB820288). The *16S rRNA* gene sequences of “*Ca. M*. haemomacaque” from this current study were deposited in GenBank under accession numbers: MW040084-MW040119.

### Phylogenetic analysis

**Figure-2:**
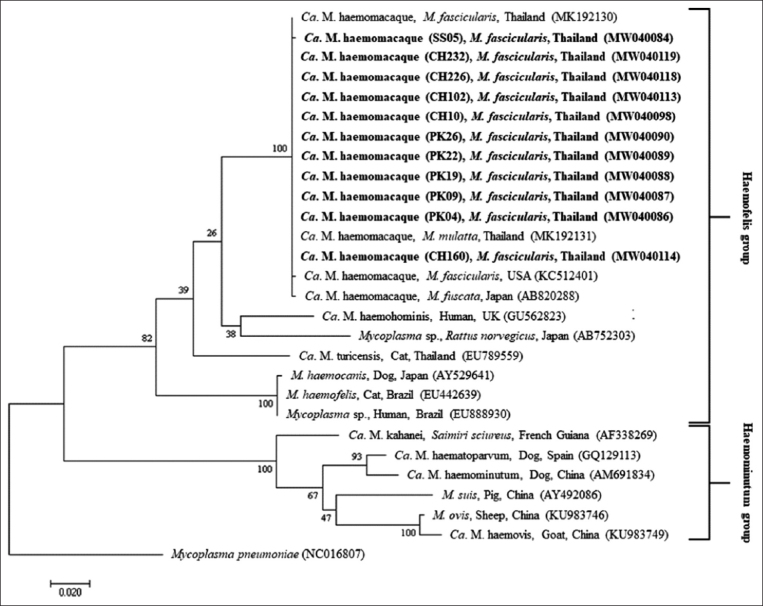
Phylogenetic tree based on 16S rRNA gene sequences of *Mycoplasma* genus produced using maximum likelihood method. Sequences detected in the current study are in bold.

A maximum-likelihood phylogenetic analysis based on the *16S rRNA* gene was performed using randomly selected positive samples from this current study, together with previously published sequences from the GenBank database. The phylogenetic tree revealed two distinct clades of hemoplasmas: The haemofelis group and the haemominutum group. The hemoplasmas detected in this current study were clustered in the haemofelis group and were grouped in the same clade with the “*Ca. M*. haemomacaque” previously detected in Japanese macaques (*M. fuscata*) from Japan (AB820288), rhesus macaques (*M. mulatta*) from Thailand (MK192131), and long-tailed macaques (*M. fascicularis*) from the USA (KC512401) and Thailand (MK192130) (Figure-2).

## Discussion

The results of this investigation demonstrate the occurrence and genetic diversity of hemoplasmas in free-ranging long-tailed macaques in the east and south of Thailand. The prevalence of “*Ca. M*. haemomacaque” infection in long-tailed macaques in this current study was 11.2%, which was lower than previously reported occurrences in the USA (84.6%, 44/52) and Thailand (55.1%, 125/227) [16,17]. Besides long-tailed macaques, “*Ca. M*. haemomacaque” has been detected in 100% (9/9) of *M. fuscata* in Japan [17] and 65.7% (23/35) of *M. mulatta* in Thailand [17].

In this study, no significant differences were noted in terms of the prevalence of hemoplasma according to animal gender and location of the macaques. However, a previous study showed that the prevalence of hemoplasma infection in female long-tailed macaques was higher than that in male long-tailed macaques from Thailand [17]. Conversely, a higher prevalence of hemoplasma infection in male macaques was reported in non-human primates in Brazil, which was possibly due to transmission by the aggressive behavior of male animals [19].

Phylogenetic analysis indicated that the *16S rRNA* sequences detected in the long-tailed macaques in this current study were clustered into the group “*Ca. M*. haemomacaque,” which was similar to the sequences detected in *M. fuscata* in Japan [18] and *M. fascicularis* and *M. mulatta* in Thailand [17]. Moreover, the occurrence of other hemoplasmas among different non-human primate species around the world has been reported, including “*Ca. M*. kahanei” in squirrel monkeys and howler monkeys [13,14] and “*Ca. M*. aoti” in owl monkeys [15].

A few previous studies described the occurrence of veterinary hemoplasma species as zoonotic agents, including a *M. haemofelis*-like species in Brazil and *M. suis* in China [4,21]. The presence of the same infective hemoplasma species in different hosts suggests its transmission to other species. Although there are no reports of macaque hemoplasmas in humans, continued investigations into the epidemiology of hemoplasmas in non-human primates, including *Ca. M*. haemomacaque, are important, as the hemoplasmas might also eventually infect humans. In addition, surveys of hemoplasmas among humans in the same environment as macaques are also necessary.

The natural transmission routes of hemoplasma in non-human primates remain to be unclear. A previous study described the presence of hemoplasma DNA in *Amblyomma* spp. and in lice from rats [3]. However, a limited number of reports describe the vectors of hemoplasmas among non-human primates. Therefore, to reduce the spread of hemoplasma among macaques, additional epidemiological studies are recommended to identify the reservoirs and vectors of hemoplasma in Thailand. In addition, further studies should include investigations of hemoplasmas in various species of non-human primates in other areas in Thailand, as well as in captive monkeys, to improve our understanding of the transmission of these parasites.

## Conclusion

As per our findings, it was determined that long-tailed macaques in Thailand can be infected and serve as reservoirs for hemoplasmas. Sequencing and phylogenetic analysis of the *16S rRNA* gene revealed that all hemoplasmas detected in the macaques in this study were “*Ca. M*. haemomacaque,” suggesting that the same strain is circulating among long-tailed macaques in many areas in Thailand.

## Authors’ Contributions

TI designed the study, revised and finalized the manuscript for submission. WS designed and conducted the study, interpreted the results, and drafted the manuscript. SuK supervised the sample collection. RR, BM, and SW assisted the sample collection. SaK and NI supervised the molecular analyses. TJ interpreted the results. All authors read and approved the final manuscript.

## References

[1] Messick J.B (2004). Hemotrophic mycoplasmas (hemoplasmas):A review and new insights into pathogenic potential. Vet. Clin. Pathol.

[2] Citti C, Blanchard A (2013). Mycoplasmas and their host:Emerging and re-emerging minimal pathogens. Trends Microbiol.

[3] Gonçalves L.R, Herrera H.M, Nantes W.A.G, Santos F.M, Porfírio G.E.O, Barreto W.T.G, de Macedo G.C, Assis W.O, Campos J.B.V, da Silva T.M.V, Mariano L.C, Barros-Battesti D.M, Machado R.Z, André M.R (2020). Genetic diversity and lack of molecular evidence for hemoplasma cross-species transmission between wild and synanthropic mammals from Central-Western Brazil. Acta Trop.

[4] dos Santos A.P, dos Santos R.P, Biondo A.W, Dora J.M, Goldani L.Z, de Oliveira S.T, de SáGuimarães A.M, Timenetsky J, de Morais H.A, González F.H.D, Messick J.B (2008). Hemoplasma infection in HIV-positive patient, Brazil. Emerg. Infect. Dis.

[5] Steer J.A, Tasker S, Barker E.N, Jensen J, Mitchell J, Stocki T, Chalker V.J, Hamon M (2011). A novel hemotropic *Mycoplasma* (hemoplasma) in a patient with hemolytic anemia and pyrexia. Clin. Infect. Dis.

[6] Hasiri M.A, Sharifiyazdi H, Moradi T (2016). Molecular detection and differentiation of canine hemoplasma infections using RFLP-PCR in dogs in Southern Iran. Vet. Arch.

[7] de Sousa K.C.M, Herrera H.M, Secato C.T, Oliveira A.D.V, Santos F.M, Rocha F.L, Barreto W.T.G, Macedo G.C, de Andrade Pinto P.C.E, Machado R.Z, Costa M.T, André M.R (2017). Occurrence and molecular characterization of hemoplasmas in domestic dogs and wild mammals in a Brazilian Wetland. Acta Trop.

[8] Do T, Kamyingkird K, Bui L.K, Inpankaew T (2020). Genetic characterization and risk factors for feline hemoplasma infection in semi-domesticated cats in Bangkok, Thailand. Vet. World.

[9] Kaewmongkol S, Lakhana N, Sirinarumitr T, Fenwick S.G, Kaewmongkol G (2020). Investigation of hemotropic *Mycoplasma* spp genotypes in client-owned cats in Thailand. Vet. Microbiol.

[10] Seo M.G, Kwon O.D, Kwak D (2019). Prevalence and phylogenetic analysis of hemoplasma species in domestic pigs in Korea. Parasit. Vectors.

[11] Wang X, Cui Y, Zhang Y, Shi K, Yan Y, Jian F, Zhang L, Wang R, Ning C (2017). Molecular characterization of hemotropic mycoplasmas (*Mycoplasma ovis* and '*Candidatus Mycoplasma* haemovis') in sheep and goats in China. BMC Vet. Res.

[12] Santos N.J.R, Brito D.R.B, Abate H.L, Paixão S.F, Soares E.D.S, Vieira T.S.W, Garcia J.L, Vieira R.F.C, Vidotto O (2018). Hemotropic mycoplasmas infection in water buffaloes (*Bubalus bubalis*) from Northeastern Brazil. Comp. Immunol. Microbiol. Infect. Dis.

[13] Bonato L, Figueiredo M.A.P, Gonçalves L.R, Machado R.Z, André M.R (2015). Occurrence and molecular characterization of *Bartonella* spp and hemoplasmas in neotropical primates from Brazilian Amazon. Comp. Immunol. Microbiol. Infect. Dis.

[14] de Melo C.M.F, Daneze E.R, Mendes N.S, de Souza Ramos I.A, Morales-Donoso J.A, Fernandes S.J, Machado R.Z, André M.R, da Rosa Sobreira M.F (2019). Genetic diversity and hematological and biochemical alterations in *Alouatta* primates naturally infected with hemoplasmas in Brazil. Comp. Immunol. Microbiol. Infect. Dis.

[15] Barker E.N, Helps C.R, Neimark H, Peters I.R, Peters W, Tasker S (2011). A novel haemoplasma species identified in archived primate blood smears. Vet. Microbiol.

[16] Maggi R.G, Mascarelli P.E, Balakrishnan N, Rohde C.M, Kelly C.M, Ramaiah L, Leach M.W, Breitschwerdt E.B (2013). *Candidatus Mycoplasma* haemomacaque and *Bartonella quintana* bacteremia in cynomolgus monkeys. J. Clin. Microbiol.

[17] Suksai P, Kaewchot S, Sereerak P, Boonnan S, Phimsin B, Jaruwattananon T, Raschasin K, Kaewparuehaschai M, Siriphet S, Bhusri B (2019). Molecular identification of hemoplasmas in free-ranging non-human primates in Thailand. Asian Pac. J. Trop. Med.

[18] Sashida H, Suzuki Y, Rokuhara S, Nagai K, Harasawa R (2013). Molecular demonstration of hemotropic mycoplasmas in wild Japanese monkeys (*Macaca fuscata*). J. Vet. Med. Sci.

[19] Cubilla M.P, Santos L.C, de Moraes W, Cubas Z.S, Leutenegger C.M, Estrada M, Vieira R.F.C, Soares M.J, Lindsay L.L, Sykes J.E, Biondo A.W (2017). Occurrence of hemotropic mycoplasmas in non-human primates (*Alouatta caraya*, *Sapajus nigritus* and *Callithrix jacchus*) of Southern Brazil. Comp. Immunol. Microbiol. Infect. Dis.

[20] Santos L.C, Cubilla M.P, de Moraes W, Cubas Z.S, Oliveira M.J, Estrada M, Leutenegger C.M, Sykes J.E, Lindsay L.L, Marcondes M (2013). Hemotropic *Mycoplasma* in a free-ranging black howler monkey (*Alouatta caraya*) in Brazil. J. Wildl. Dis.

[21] Yuan C.L, Liang A.B, Yao C.B, Yang Z.B, Zhu J.G, Cui L, Yu F, Zhu N.Y, Yang X.W, Hua X.G (2009). Prevalence of *Mycoplasma suis* (*Eperythrozoon suis*) infection in swine and swine-farm workers in Shanghai, China. Am. J. Vet. Res.

[22] Roos C, Zinner D (2015). Diversity and evolutionary history of macaques with special focus on *Macaca mulatta* and *Macaca fascicularis*. In:The Nonhuman Primate in Nonclinical Drug Development and Safety Assessment. Elsevier, Amsterdam.

[23] Kaewmongkol G, Maneesaay P, Suwanna N, Tiraphut B, Krajarngjang T, Chouybumrung A, Kaewmongkol S, Sirinarumitr T, Jittapalapong S, Fenwick S (2016). First detection of *Ehrlichia canis* in cerebrospinal fluid from a nonthrombocytopenic dog with meningoencephalitis by broad-range PCR. J. Vet. Intern. Med.

